# Phenylketonuria: nutritional advances and challenges

**DOI:** 10.1186/1743-7075-9-7

**Published:** 2012-02-03

**Authors:** Marcello Giovannini, Elvira Verduci, Elisabetta Salvatici, Sabrina Paci, Enrica Riva

**Affiliations:** 1 Department of Pediatrics, San Paolo Hospital, University of Milan, Milan, Italy

**Keywords:** PKU, treatment advances, new strategies, LCPUFA supplementation, LNAA, sapropterin

## Abstract

Despite the appearance of new treatment, dietary approach remains the mainstay of PKU therapy. The nutritional management has become complex to optimize PKU patients' growth, development and diet compliance. This paper review critically new advances and challenges that have recently focused attention on potential relevant of LCPUFA supplementation, progress in protein substitutes and new protein sources, large neutral amino acids and sapropterin. Given the functional effects, DHA is conditionally essential substrates that should be supplied with PKU diet in infancy but even beyond. An European Commission Programme is going on to establish quantitative DHA requirements in this population. Improvements in the palatability, presentation, convenience and nutritional composition of protein substitutes have helped to improve long-term compliance with PKU diet, although it can be expected for further improvement in this area. Glycomacropeptide, a new protein source, may help to support dietary compliance of PKU subject but further studies are needed to evaluate this metabolic and nutritional issues. The PKU diet is difficult to maintain in adolescence and adult life. Treatment with large neutral amino acids or sapropterin in selected cases can be helpful. However, more studies are necessary to investigate the potential role, dose, and composition of large neutral amino acids in PKU treatment and to show long-term efficacy and tolerance. Ideally treatment with sapropterin would lead to acceptable blood Phe control without dietary treatment but this is uncommon and sapropterin will usually be given in combination with dietary treatment, but clinical protocol evaluating adjustment of PKU diet and sapropterin dosage are needed.

In conclusion PKU diet and the new existing treatments, that need to be optimized, may be a complete and combined strategy possibly positive impacting on the psychological, social, and neurocognitive life of PKU patients.

## Introduction

Since the first attempt to treat a patient affected by phenylketonuria (PKU) with a low phenylalanine (Phe) diet, many steps forward have been made in the treatment of PKU. Progress in the development of alternative approaches to the treatment of PKU has been very impressive over the past decade (large neutral amino acids, sapropterin and more recently phenilalanine ammonia lyase or gene therapy). Nevertheless, since nutritional intervention remains essential in the management if this disease, we must be aware of the new advances and challenges in the nutritional management of PKU. This paper review critically new advances and challenges that have recently focused attention on potential relevant of long chain polyunsaturated fatty acids (LCPUFA) supplementation, progress in protein substitutes and new protein sources, large neutral amino acids, sapropterin and phenilalanine ammonia lyase.

### Goals of dietary treatment

The main goals of treatment for PKU is to maintain the blood Phe levels within safe limits (i.e. ranging 120-360 umol/L, for pregnant women 120-240 umol/L), to prevent mental retardation, to ensure normal growth and normal life with good health through adulthood. This can be done by introduction of a low-Phe diet. When started in the neonatal period it modifies the metabolic phenotype and prevents the neuropsychological consequences of hyperphenylalaninemia [1]. Indeed it is known that high blood Phe levels are neurotoxic, mainly for its inhibitory action on the transport of free L-amino acids (leucine, isoleucine, valine, tyrosine, tryptophan, and lysine), necessary for the protein and neurotransmitters synthesis (dopamine and serotonin). The aim of the dietary treatment is to avoid acute and chronic increased concentrations of Phe in plasma and consequently in cerebral tissue, responsible for a important worsening of the neuronal performance and the behaviour [1]. Prognosis and outcome strongly depend on the age when the diagnosis is made and treatment introduced, but also on the type of mutation. Because of evidence that early ending of treatment might impair later neuropsychological performance, the treatment of PKU is recommended for life [1]. Compliance with treatment is adequate in infancy and childhood, however difficulties in maintaining a PKU diet in adolescent and adulthood are reported [2]. Based on a survey of 10 European centres, Ahring et al. [3] concluded that on average only in 65% of samples from patients above 16 years of age Phe values were within or below guideline goals.

Although elevated Phe concentrations seem to have smaller influence on brain functioning beyond early childhood [4], there is evidence that numbers of neuropsychological and neurophysiological dysfunctions can appear in older patients, as the consequence of losing metabolic control [2] and that may be associated to subsequent poor school and work performance [2]. Although severe neurological damage is completely prevented in PKU affected individuals by adequate dietary therapy, subtle neurological deficits persist and are detectable. For example, the IQ of well and early treated PKU children participating in the German Collaborative Study was 3-6 points lower than that of their healthy siblings [5]. Moreover in this study was observed that after early and strict treatment during the preschool years, variation of observed Phe did not influence IQ development at school age. Thus, other factors than the degree of Phe control appear to influence cognitive outcomes. In another study, early and continuously treated PKU patients of ages 8 to 20 years were compared with controls with respect to behaviour and school achievements [6]. PKU patients demonstrated more problems in task-oriented behaviour and average academic performance than controls.

### PKU diet: nutritional characteristics

The restriction of dietary phenylalanine remains the mainstay of PKU management and usually begins immediately after confirmation of this condition in a newborn. Patients with PKU have to avoid foods rich in protein (meat, fish, eggs, standard bread, dairy products, nuts, and seeds) and foods and drinks containing aspartame, flour, soya, beer, or cream liqueurs. Therefore PKU diet is mainly made up by low-protein natural foods (vegetables, fruits and some cereals), poor in Phe, to reach the Phe supply necessary for growth processes. Low-protein variants of some foods are commercially present, such as low-protein bread and low-protein pasta. The quantities of the permitted natural foods are calculated from a method of Phe equivalence (dependent upon how much Phe a food contains for a given weight). The required amount of daily protein is obtained from Phe-free protein substitutes providing essential amino acids in suitable proportions.

During infancy breastfeeding or human milk meals [7], to provide natural protein according to the individual Phe tolerance, and a Phe-free infant formula are recommended.

This type of dietary regimen, provide lower saturated and polyunsaturated fat, cholesterol, carnitine taurine, iron, zinc, selenium, calcium, folates, A, C, D, E and B2, B6, B12 vitamins intake, because of the very low assumption of Phe-containing animal foods, as well as higher carbohydrates intake than healthy pediatric population. Therefore there is a general agreement that patients with PKU need long-term dietary counseling and daily micronutrients supplementation. However some advantages of this dietary treatment should be considered. The PKU diet follows the norms of the so-called "prudent" diet for the prevention of cardiovascular disorders. In particular saturated fats may be less than 7% and polyunsaturated higher than 5% total energy with a supply less than 50 mg cholesterol per day [8]. Indeed PKU children show lower plasma cholesterol levels as compared to healthy children, particularly low density lipoprotein particles [9]. Nevertheless both dietary habits and genetic predisposition (apo B and apo E polymorphisms) may interact in keeping low blood lipid levels in PKU population [9].

### PKU: nutritional advances and challenges

#### PKU and LCPUFA

##### Rationale for LCPUFA supplementation

PKU children who are compliant with the recommended low protein diet are devoid of natural dietary sources of n-3 LCPUFA, as LCPUFA rich foods also contain high amounts of protein. Accordingly, blood concentrations of n-3 LCPUFA and especially of docosahexaenoic acid (DHA) are reduced in plasma and red blood cell phospholipids of PKU children relative to omnivorous children [10-12] with a more marked relative depletion of DHA than arachidonic acid, the major n-6 LCPUFA, throughout childhood. LCPUFA with 20 and 22 carbon atoms are metabolically derived from α-linolenic acid (ALA) and linoleic acid (LA) by consecutive enzymatic desaturation and chain elongation. Although LCPUFA including DHA (22:6n-3) and AA (20:4n-6) can be synthesized from ALA and LA in mammals, respectively, the activity of conversion is low in humans particularly for the synthesis of DHA, which is formed through a far more complex and indirect metabolic pathway than AA [13]. Blood levels of AA are influenced by polymorphisms of the fatty acid desaturase genes to a far greater degree than DHA levels (around 28-30% vs 2-3%) [14], which also points towards a low contribution of endogenous n-3 conversion to DHA and the importance of a preformed dietary intake of DHA. There is increasing awareness of the importance of DHA for optimal neurological function in humans. DHA status during prenatal and postnatal development has been linked to benefits for child outcomes in several studies [15]. In particular the striking variations that have been found in the DHA content of prefrontal brain cortex in breastfed compared with formula-fed infants provide convincing evidence of the effects of a dietary supply of LCPUFA on the structure of the brain [16]. The extremely low dietary DHA intake in PKU children, with resulting low blood and presumably tissue DHA contents, may have adverse consequences for central nervous function, which might contribute to learning difficulties, behavioral abnormalities and visual problems found in PKU patients [17-19]. Early dietary treatment of PKU prevents severe neurological damage and generally leads to normal cognitive development, but subtle neurological deficits persist [5]. Poorer school performance of PKU children was reported, with significantly lower academic performance, initiative and progress than in age matched omnivorous children [6]. These subtle functional deficits in early and well treated individuals with PKU are not explained by variations in plasma Phe levels, and it is plausible that a low DHA supply induced by the extremely restricted diet contributes to neurological abnormalities.

##### LCPUFA supplementation: infancy and childhood

Because the first few months of life represent the most vulnerable period for brain development, early supplementation with preformed LCPUFA could be an important addition to the nutrition of infants with PKU [15]. A double-blind randomized trial [20] showed that a dietary supply of LCPUFA in infants with PKU (a Phe-free formula supplemented with DHA, 0.3 g/100 g fatty acids, and AA, 0.7 g/100 g) prevents the decline in DHA status associated with a diet supplying minimal sources of LCPUFA (breast milk).

Therefore by using a phenylalanine-free infant formula supplemented with LCPUFA in the first year of life, the risk that infants with PKU will have sub-optimal DHA status may be minimized.

Hovewer, LCPUFA supplementation is not routinely provided in PKU after infancy. In PKU children one double-blind, placebo-controlled randomised trial was conducted to elucidate the biochemical and functional effects of LCPUFA supplementation [21]. Twenty children with PKU received supplementation either with a fat blend balanced in both n-3 and n-6 LCPUFA (providing about 8 mg eicosapentaenoic acid, EPA, and 10 mg DHA per Kg body weight per day) or placebo for 12 months (olive oil). Both n-3 and n-6 fatty acids were supplemented to avoid biochemical imbalances. By the end of the trial plasma levels of these fatty acids raised and the latency time in the P100 wave of visually evoked potentials (VEP) decreased significantly in children receiving the preparation with LCPUFA. However, 3 years after supplementation, no biochemical or functional differences between supplemented and unsupplemented children were apparent [22]. In 2 open clinical trials [23,24], also including a few children younger than 2 years, a 3 month supplementation of patients with classical PKU with fish oil providing EPA and DHA (15 mg of DHA per kg of body weight per day) without major amount of n-6 LCPUFA, significantly increased n-3 LCPUFA levels in plasma phospholipids, but also significantly decreased the arachidonic acid level, and shorted the latencies of VEP and improved the performance in the Rostock-Oseretzky Scale for coordination and fine motor skills [24].

In conclusion given the functional effects, DHA is conditionally essential substrates that should be supplied with PKU diet even beyond infancy. Their addition to the synthetic amino acid mixtures appears advisable.

##### LCPUFA supplementation: dosage

The dosage used in Beblo study [24] resulted in supra-normal plasma n-3 LCPUFA and reduced AA levels, hence the daily dose of 15 mg DHA/Kg may be higher than the dose needed for oprimal outcomes. In addition to a required confirmation of the observed DHA effects in a further study, quantitative needs should be established [25]. With financial support provided by the 7^th ^Framework Research Programme of the European Commission, a multicentre randomized clinical trial in school age PKU children is currently going on (NUTRIMENTHE EU Project "Effect of Diet on Mental Performance of Children" FP7-KBBE-2007-1, GA: 212652). PKU children, school age, will be randomised to receive different dosage of DHA supplement (0-15 mg/Kg/day) and will be tested for functional outcomes both before and after supplementation [25]. This trial may achieve to establish quantitative DHA requirements in children and thus may allow conclusions on the desirable dietary DHA supply to healthy children, since there are no indications that PKU-children have any alterations of energy and fatty acid metabolism, and of DHA absorption, distribution, metabolism and excretion.

##### LCPUFA supplementation: other paediatric and adult PKU related population

Pregnant women with PKU and poorly compliant patients with the condition also might benefit from preformed LCPUFA supplementation. Supplementation of pregnant women should help assure maximal placental transfer of these fatty acids during the last 3 months of pregnancy. According to international recommendations, healthy pregnant woman should consume preformed n-3 LCPUFA providing an average intake of at least 200 mg DHA/day on a regular basis [15]. Moreover supplementation of poorly compliant patients should provide higher levels of neuroprotection and lessen the theoretical risk of inhibition of endogenous LCPUFA synthesis by toxic phenylalanine by-products. In fact, it has been observed that [17], in the patients with inadequate metabolic control, the increased concentrations of Phe and of its metabolites, phenylpiruvic and fenyllactic acids, can inhibit for competition the synthesis of an important enzymatic cofactor, the α-tocopherolchinone, which facilitates the action of the desaturase in the synthesis process of fatty acids.

Studies are needed to evaluate the optimal dosage of DHA supplementation in PKU pregnancy and in PKU poorly compliant patients.

#### Progress in the dietary treatment: protein substitutes and new protein sources

##### Protein substitutes

The number of protein substitutes (mixtures of amino acids that are free from or low in Phe) available for PKU patients is increasing constantly with time [26]. The absence of Phe is the one constant in these mixtures; there is variability in the presence or absence of lipids, vitamins and minerals with differences also in the amount.

The major progresses in protein substitutes are:

⇒ Palatability and caloric content: better taste of amino acid powder/lower calories (lipid and carbohydrate composition of preparations have been improved);

⇒ Compliance (all ages): better compliance due to different formulations (powder, tablets, shake, creams etc)

⇒ Age-related problems with diet (childbearing age, adolescence, adult age,): different age-related formulas and composition → individualized dietary treatment.

Improvements in the palatability, presentation, convenience and nutritional composition of substitutes have helped to improve long-term compliance with PKU diet, although it can be expected for further improvement in this area. About the composition of the mixtures according the different age we could consider:

1) Phe-free mixtures of amino acids for infants 0-12 month: the composition of each products meets infants' requirements. Most of the different protein substitutes contain DHA and AA and some formulations are supplemented with prebiotics.

2) Phe-free mixtures of amino acids for children, adolescents and adults: many products are available with different composition and presentation. This represents a great advantage for patients as they can choose the product most likely to meet their needs.

Nevertheless the use of protein substitutes should continue to be observed and monitored the efficacy proven in long-term studies.

##### New protein sources: glycomacropeptide

Glycomacropeptide (GMP) is a protein derived from cheese whey that is rich in specific essential aminoacids but it is the only known natural protein that in its purified form is free of tyrosine, tryptophan and phenylalanine (aromatic aminoacids) and therefore could provide an alternative protein source for PKU individuals when manufactured to sufficient purity to ensure it is free from Phe that could be contained in traces of other whey proteins and with supplementation of missing aromatic amino acids other than Phe [27]. In the last years progresses in evaluating the safety, acceptability and efficacy of GMP in the nutritional management of PKU have been performed [28]. A variety of foods and beverages can be made with GMP to improve the taste, variety and convenience of the PKU diet. Sensory studies in individuals with PKU demonstrate that GMP foods are acceptable alternatives to amino acid medical foods. Studies in the PKU mouse model demonstrate that GMP supplemented with limiting indispensable amino acids (tyrosine and tryptophan) provides a nutritionally adequate source of protein and improves the metabolic phenotype by reducing concentrations of Phe in plasma and brain [29]. In a recent study on 11 PKU patients the GMP diet was preferred to the amino acid diet [30]. When comparing fasting with postprandial plasma, plasma Phe concentration increased significantly with the amino acid diet but not with the GMP diet. Blood urea nitrogen was significantly lower, which suggests decreased urea genesis, and plasma insulin was higher with the GMP diet than with the amino acid diet. The authors concluded that GMP, when supplemented with limiting amino acids, is a safe and highly acceptable and may improves protein retention and Phe utilization compared with amino acid diet. A more recent study assess the ability of a GMP breakfast to promote satiety in 11 PKU subjects (8 adults and 3 boys ages 11-14), when compared to an amino acid-based breakfast [31]. Postprandial ghrelin concentration was significantly lower with GMP, associated with greater feelings of fullness after breakfast, compared to an amino acid-based breakfast, with no difference in fasting ghrelin. Postprandial concentrations of insulin and total plasma amino acids were higher after a GMP breakfast compared to an amino acids-based breakfast consistent with slower absorption and less degradation of amino acids from GMP.

In conclusion GMP may help to support dietary compliance of PKU subject but further studies are needed to evaluate this metabolic and nutritional issues.

#### New strategies

The PKU diet is difficult to maintain in adolescence and adult life. Many adults discontinue it or refuse to return to it, which is a continuous challenge in the follow-up of PKU. Treatment with large neutral aminoacid (LNAA) supplements or tetrahydrobiopterin (BH4) cofactor in could be helpful.

##### Large neutral amino acids

Apart from Phe, LNAAs include tyrosine, tryptophan, threonine, methionine, valine, isoleucine, leucine, and histidine. In healthy individuals, all of these except tyrosine are essential amino acids. In patients with PKU, tyrosine has become an essential amino acid. LNAA treatment as an alternative to dietary Phe restriction was suggested as early as 1953 [32]. Since then, different combinations of LNAAs have been designed, based on different rationales and treatment targets. Recently the use of LNAAs as an alternative treatment in PKU have been reviewed [33]. LNAAs supplementation may have multiple treatment targets: a specific reduction in brain phenylalanine concentrations, a reduction in blood (and consequently brain) phenylalanine concentrations, an increase in brain neurotransmitter concentrations, and an increase in brain essential amino acid concentrations. Although LNAA treatment in PKU was initially intended to be unrestricted natural protein intake combined with LNAA supplementation, it should be noted that not all authors studied LNAA supplementation in this manner. Pietz et al. [34] confirmed that the LNAA blocked the Phe transport into the brain by giving an oral ''Phe challenge'' with and without LNAA and measuring the influx of Phe into the brain. The use of the LNAA therapy has been shown to improve amino acid profiles as well as increase tyrosine and tryptophan concentrations in the blood, which are precursors for dopamine and serotonin [35]. A short-term double-blind placebo control study, using LNAA in patients with PKU, showed a lowering of blood Phe concentration by an average of 39% from baseline [36]. Additionally LNAA treatment seems to have a beneficial effects on executive functioning [37].

In conclusion, LNAA treatment is seen as an alternative to conventional dietary PKU treatment. LNAA treatment may refer to at least two different LNAA treatment strategies, i.e., natural protein intake at Recommended Dietary Allowance (RDA) with LNAA supplementation, and conventional dietary therapy combined with LNAA supplementation. In addition, LNAA treatment may refer to supplementation of single amino acids, such as tyrosine, tryptophan, and threonine. These differences in treatment strategies are based on clearly different theories regarding mechanism of action. Consequently, more studies are necessary to investigate the potential role, dose, and composition of LNAA in PKU treatment and to show long-term efficacy and tolerance.

##### Sapropterin dihydrochloride supplementation in BH4 responsive PKU patients

Long-term adherence (and hence metabolic control) to PKU diet is commonly poor. Some phenylalanine hydroxylase (PAH) mutations are associated with a BH4-sensitive phenotype of PKU, in which giving pharmacological doses of exogenous BH4 results in an increase in the activity of PAH [38]. These mutations usually present with substantial residual activity. Indeed response to BH4 is usually more pronounced in mild or moderate PKU. Patients with PKU (usually, but not exclusively, with a relatively mild form of the disorder) who are responsive to treatment with pharmacological doses of BH4 have either lower concentrations of blood phenylalanine or improved dietary phenylalanine tolerance [2,39]. The availability of a registered formulation of BH4 (sapropterin dihydrochloride) has raised many practical issues and new questions in the dietary management of these patients [40]. Approssimately 20-60% of patients have been showen to achieve a > 30% reduction in blood Phe levels with sapropterin or previous unregistered formulation of BH4 [40]. Ideally treatment with sapropterin would lead to acceptable blood Phe control without dietary treatment but this is uncommon and sapropterin will usually be given in combination with dietary treatment.

##### Sapropterin and PKU diet

Initially, patients and carers must understand clearly the likely benefits (and limitations) of sapropterin therapy. A minority of patients who respond to sapropterin are able to discontinue the phenylalanine-restricted diet completely, while others are able to relax the diet to some extent. Care is required when altering the phenylalanine-restricted diet, as this may have unintended nutritional consequences and must be undertaken with caution. New clinical protocols are required for managing any dietary change while maintaining control of blood phenylalanine, ensuring adequate nutrition and preventing nutritional deficiencies, overweight or obesity. An accurate initial evaluation of pre-sapropterin phenylalanine tolerance is essential, and the desired outcome from treatment with sapropterin (e.g. reduction in blood Phe or relaxation in diet) must also be understood by the patient and carers from the outset. Continuing education and support will be required thereafter, with further adjustment of diet and sapropterin dosage as a young patient grows. Multicentric standardised long-term studies are necessary to assess the nutritional adequacy, growth, body composition, blood Phe control during illness, diurnal blood Phe variability and changes in the quality and variety of foods eaten. Guidance is needed about the amount and type of natural protein that should be consumed before protein substitute is stopped and how best to introduce dietary Phe with sapropterin. In addition only data for the effect on Phe metabolism are available. Data for neurocognitive outcomes in these patients are scarce [41] and little is known about longer-term adherence to sapropterin treatment. New protocols are required for managing dietary treatment in association to use of sapropterin.

##### Phenylalanine ammonia lyase

Phenylalanine ammonia lyase (PAL) is a plant-derived enzyme that also degrades phenylalanine (without synthesizing tyrosine), but it is more stable than PAH and does not require a cofactor. Pegylated PAL (PEG-PAL) is a novel formulation of PAL that is currently undergoing clinical trials. Recently in a mouse model of phenylketonuria [42], the PEG-PAL, given orally, showed promising results with a mean blood phenylalanine level reduction of around 40% and no serious adverse reactions. Phe reduction occurred in a dose- and loading-dependent manner. In conclusion oral PAL therapy could be an adjunct therapy, perhaps with dietary treatment, independently from the PAH genotype.

## Conclusion

In PKU severe neurological and functional disorders can be prevented through an early strict nutritional treatment aimed at reducing blood Phe to non-toxic levels through a PKU diet. The outcome for patients with PKU treated from the first weeks of life is generally excellent with children attaining developmental milestones and attending normal schools. Significant clinical psychological and neurocognitive abnormalities are observed in patients non-compliant with PKU.

New advances and challenges has been performed in the nutritional management of PKU (i.e. LCPUFA supplementation, progress in protein substitutes and new protein sources). Long term dietary guidance and monitoring of the nutritional status of patients with PKU should be part of a follow-up programme that continues for life. Pediatricians and dieticians should prescribe and carefully monitor macronutrient and micronutrient intake, growth and physical activity in PKU patients. New treatments exist-such as LNAA and sapropterin -, that need to be optimized as far as targets, expected outcomes and nutritional follow up. In particular a clinical protocol evaluating adjustment of PKU diet and sapropterin dosage are needed. Therefore despite the appearance of new advances and strategies, dietary treatment remains the mainstay of PKU therapy. Moreover the new treatment options (LNAA, sapropterin, PAL) for the management of PKU are expanding, and do not always allow a complete relaxation of PKU diet. Therefore the nutritional treatment is always essential. However it is necessary evaluate if PKU diet and the new treatment options in combination may really matter in the psychological, social, and neurocognitive life of PKU patients. Anyway a complete strategy should include anyway long term dietary guidance and monitoring of the nutritional status of PKU patients.

## Abbreviations lists

AA: arachidonic acid; ALA: alpha-linolenic acid; BH4: tetrahydrobiopterin; CNS: central nervous system; DHA: docosahexaenoic acid; GMP: glycomacropeptide; HPA: hyperphenylalaninemia; IQ: intelligence quotient; LA: linoleic acid; LCPUFA: long chain polyunsaturated fatty acids; LNAA: large neutral amino acids; MHP: mild hyperphenylalaninaemia; PAH: phenylalanine hydroxylase; Phe: phenylalanine; PKU: phenylketonuria; PUFA: polyunsaturated fatty acids; RDA: recommended daily allowance; Thr: threonine; Tyr: tyrosine; VEP: visual evoked potentials.

## Competing interests

The authors declare that they have no competing interests.

## Authors' contributions

MG had primary responsibility for review development, coordinated the research team, supervised the manuscript. EV, had primary responsibility for references searching and wrote the manuscript. ES, participated in references searching, and contributed to the writing of the manuscript. SP, contributed to the writing of the manuscript. ER participated in the development of the review and contributed to the writing of the manuscript. All authors read and approved the final manuscript.
